# Association between osteocalcin, a pivotal marker of bone metabolism, and secretory function of islet beta cells and alpha cells in Chinese patients with type 2 diabetes mellitus: an observational study

**DOI:** 10.1186/s13098-022-00932-8

**Published:** 2022-10-28

**Authors:** Haiyan Lei, Jun Liu, Wei Wang, Xinyi Yang, Zhouqin Feng, Pu Zang, Bin Lu, Jiaqing Shao

**Affiliations:** 1 Department of Endocrinology, Jinling Hospital, The First School of Clinical Medicine, Southern Medical University, 305 East Zhongshan Road, Nanjing, 210002 Jiangsu People’s Republic of China; 2 Department of Endocrinology, Jinling Hospital, School of Medicine, Nanjing University, Nanjing, 210002 People’s Republic of China

**Keywords:** Serum osteocalcin, Blood glucose, Pancreatic secretion, Glucagon secretion, Type 2 diabetes mellitus

## Abstract

**Background:**

Several recent studies have found that Osteocalcin (OCN), a multifunctional protein secreted exclusively by osteoblasts, is beneficial to glucose metabolism and type 2 diabetes mellitus (T2DM). However, the effects of OCN on islets function especially islet ɑ cells function in patients with type 2 diabetes mellitus characterized by a bi-hormonal disease are still unclear. The purpose of this cross-sectional study was to investigate the relationship between serum OCN and the secretion of islet β cells and ɑ cells in Chinese patients with type 2 diabetes mellitus.

**Methods:**

204 patients with T2DM were enrolled. Blood glucose (FBG, PBG0.5h, PBG1h, PBG2h, PBG3h), insulin (FINS, INS0.5h, INS1h, INS2h, INS3h), C-peptide (FCP, CP0.5h, CP1h, CP2h, CP3h), and glucagon (GLA0, GLA0.5 h, GLA1h, GLA2h, GLA3h) levels were measured on 0 h, 0.5 h, 1 h, 2 h, and 3 h after a 100 g standard bread meal load. Early postprandial secretion function of islet β cells was calculated as Δcp0.5h = CP0.5-FCP. The patients were divided into low, medium and high groups (T1, T2 and T3) according to tertiles of OCN. Comparison of parameters among three groups was studied. Correlation analysis confirmed the relationship between OCN and pancreatic secretion. Multiple regression analysis showed independent contributors to pancreatic secretion.

**Main results:**

FBG, and PBG2h were the lowest while Δcp0.5h was the highest in the highest tertile group (respectively, p < 0.05). INS3h, area under the curve of insulin (AUC_ins3h_) in T3 Group were significantly lower than T1 Group (respectively, p < 0.05). GLA1h in T3 group was lower than T1 group (p < 0.05), and GLA0.5 h in T3 group was lower than T2 and T1 groups (p < 0.05). Correlation analysis showed OCN was inversely correlated with Homeostatic model of insulin resistance (HOMA-IR), INS3h, AUC_ins3h_ (p < 0.05), and was still inversely correlated with FCP, GLA0.5 h, GLA1h, area under the curve of glucagon (AUC_gla3h_) (respectively, p < 0.05) after adjustment for body mass index (BMI) and alanine aminotransferase (ALT). The multiple regression analysis showed that OCN was independent contributor to Δcp0.5h, GLA0.5h and GLA1h (respectively, p < 0.05).

**Conclusions:**

Higher serum OCN level is closely related to better blood glucose control, higher insulin sensitivity, increased early-phase insulin secretion of islet β cells and appropriate inhibition of postprandial glucagon secretion of islet ɑ cells in adult patients with type 2 diabetes mellitus.

## Introduction

Type 2 diabetes mellitus (T2DM) is a multifaceted disease and the regulation of glucose metabolism relies on the interplay of multiple hormones that act in many target organs. In 1975 Unger et al. [1] described type 2 diabetes mellitus as a bi-hormonal disease characterized by a defective insulin secretion and hyperglucagonemia with elevated blood levels of glucose. In contrast to the effects on levels of insulin, T2DM patients with hyperglycemia often show persistent fasting hyperglucagonemia and lack of suppression of glucagon levels in the postprandial state [2, 3].

Osteocalcin (OCN) is a multifunctional protein secreted exclusively by osteoblasts [4]. There are two forms of Osteocalcin in serum, carboxylated and decarboxylated. Decarboxylated OCN is an important determinant of its metabolic activity. Due to the limited detection technology, the total serum osteocalcin level is often measured in humans. A growing number of clinical studies have observed that serum osteocalcin is associated with glucose metabolism and metabolic syndrome. OCN levels were correlated negatively with the levels of glucose, hemoglobin A1c (HbA1c), insulin resistance, body mass index (BMI), visceral fat as well as atherosclerosis parameters and cardiovascular risk factors in type 2 diabetes patients, while positively with the adiponectin levels [5–12]. What is more, osteocalcin protects against the development of metabolic diseases and type 2 diabetes mellitus [13–16]. Osteocalcin also linked to the presence or absence of diabetic kidney disease, and the presence and severity of diabetic retinopathy [16–20]. The patients with lower circulating osteocalcin concentrations exhibited higher insulin resistance and systemic inflammation (high-sensitivity C-reactive protein [hsCRP] and interleukin-6), adipokines (leptin and adiponectin), and ectopic body fat aggregation especially in young overweight and obese women [4, 7, 9, 21, 22]. Decarboxylated OCN negatively correlated with percent trunk fat, visceral/subcutaneous fat ratio (by Computed Tomography (CT)), and fat mass as well as fasting plasma glucose and HbA1c in men and postmenopausal women with T2DM [23].

In 2007, Lee et al. [24] found that decarboxylated Osteocalcin (OCN) directly increased β-cell proliferation and insulin secretion. Also, decarboxylated OCN could improve insulin resistance and protect mice from obesity and glucose intolerance. Decarboxylated OCN stimulated β-cell function and insulin production, regulated gene expression in adipocytes (including adiponectin expression), decreased visceral fat, and protected against diet-induced obesity [13]. Decarboxylated OCN significantly augmented insulin content and enhanced human β-cell proliferation [25]. Esp^−/−^ mice which is a gain-of-function model of osteocalcin activity, display increased insulin secretion, and reduced fat mass [14]. Decarboxylated OCN may play a regulatory role in various organs and tissues through different receptors, such as GPRC6A [26–28], and affect liver fat accumulation, adipose tissue distribution, gut hormones secretion, muscle motility and energy expenditure, testicular fertility, brain cognitive function and fetal brain development [4, 14, 29–33].

However, few studies have been reported whether osteocalcin affects islet ɑ cells, After 7 days of decarboxylated-OCN- treated (1.0 ng/mL) dispersed human islets, OCN significantly reduced ɑ-cell mass. Immunohistochemistry of islet grafts after infusing 4.5-ng/h decarboxylated-OCN showed islet β-cell co-expressed the ɑ-cell marker glucagon, which may explain the decrease in the number of ɑ cells [25]. However, serum levels and pancreas content of glucagon, particular the ratio of insulin-to-glucagon–positive cells were not affected in Gprc6a_Pdx1_^−/−^mice compared with control littermates [27]. Serum glucagon level was normal, not decreased [14, 24] in Esp^−/−^ mice that displayed severe hyperinsulinemia.

The skeleton exerted an endocrine regulation of glucose homeostasis through OCN. However, the effects of OCN on islets function especially islet ɑ cells function in patients with type 2 diabetes mellitus characterized by a bi-hormonal disease are still unclear. The purpose of this cross-sectional study was to investigate the relationship between serum OCN level and the secretion of islet β cells and ɑ cells in 204 Chinese patients with type 2 diabetes mellitus, and try to find a new way to prevent and treat type 2 diabetes mellitus.

## Research design and methods

### Study subjects

204 patients with T2DM hospitalized in the endocrinology wards in Jinling Hospital from September 2016 to February 2021 were enrolled. The diagnosis of T2DM was defined according to 1999 World Health Organization Criteria. Exclusion criteria were as follows: (1) taking drugs that affect the secretion of glucagon such as insulin, glucagon like peptide 1 (GLP-1)receptor agonists, and dipeptidyl peptidase-4(DPP-4) inhibitors; (2) acute complications of diabetes such as diabetic ketoacidosis; (3) severe liver and kidney dysfunction, recent acute infections and stress conditions such as cardiac insufficiency, trauma, pregnancy, or any other infectious diseases; (4) blood diseases, malignant tumors, thyroid and parathyroid diseases; (5) taking antiosteoporosis drugs (bisphosphonate, calcium, vitamin D, and vitamin K), estrogen analogs, or antineoplastic, or immune-modulating drugs within the 12 months before enrollment. All of them took a 100 g standard bread meal test. The plasma glucose, insulin, C-peptide, and glucagon levels were measured on fasting basis, and 0.5 h, 1 h, 2 h, and 3 h after a 100 g bread meal load. The patients were divided into three groups according to the tertiles of serum OCN levels, 67 cases with OCN < 14.02 ng/mL (T1 group), 68 cases with 14.02 ≤ OCN < 19.78 ng/mL (T2 group) and 69 cases with OCN ≥ 19.78 ng/mL (T3 group).

### Clinical and biochemical indexes

Venous blood sample were collected in the morning following an overnight fast. Sociodemographic characteristics, physical examination information and laboratory measurements such as gender, age, durations, body mass index (BMI), serum uric acid (SUA), blood lipid, blood pressure, liver and kidney function parameters and blood glucose were collected by trained doctors. HbA1c was determined by high performance liquid chromatography (HLC-723G8 Automatic glycosylated hemoglobin Analyzer, TOSOH, Japan). Biochemical indexes were measured by automatic biochemical analyzer (Model 7600 Series Automatic Analyzer, Hitachi, Japan). Insulin and C-peptide concentrations were measured by electricity chemiluminescent immunoassay (IMMULITE 2000 XPi, Siemens, Germany) and glucagon was measured by radioimmunoassay (RIA, Millipore, Billerica, MA, USA). Serum OCN levels were evaluated by the electrochemiluminescence immunoassay method on a Roche COBAS E 801 (Roche Diagnostics Corporation, Mannheim, Germany).

### Standard bread meal test

All subjects were fasted at least 8 h before this testing. All patients stopped all hypoglycemic drugs in the morning, and received a 100 g standard bread meal test (bread made of 100 g dry wheat white flour containing carbohydrate equals 75 g glucose. To avoid the gastrointestinal side effects of taking large amounts of glucose orally, we used the standard 100 g steamed bread meal test instead of the 75 g oral glucose tolerance test.) [34, 35] and a simultaneous insulin-C-peptide-glucagon releasing test in 3 h. Concentrations of serum glucose (FBG, PBG0.5h, PBG1h, PBG2h, PBG3h), insulin (FINS, INS0.5 h, INS1h, INS2h, INS3h), C-peptide (FCP, CP0.5h, CP1h, CP2h, CP3h) and glucagon (GLA0, GLA0.5h, GLA1h, GLA2h, GLA3h) levels were recorded at 0 h, 0.5 h, 1 h, 2 h and 3 h after meal. Areas under the curve of glucose (AUCglu3h), glucagon (AUC_gla3h_),C-peptide (AUC_cp3h_) and insulin (AUC_ins3h_) in 3 h were calculated with trapezoidal methods.

### Evaluation secretion function of pancreatic alpha cell and beta cell

Homeostatic model of insulin resistance (HOMA-IR) was calculated using the formula: [fasting insulin mIU/L) × fasting plasma glucose (mmol/L)]/22.5. Homeostasis model of β cell function (HOMA-β) = 20 × fasting insulin (mIU/L)/(fasting plasma glucose(mmol/L)-3.5); insulin and C peptide at all time points, AUC for insulin and C-peptide in 3 h (AUC_ins3h_, AUC_cp3h_) and HOMA-β were used to assess the pancreatic beta cell function. Early postprandial secretion function of islet β cells was calculated as Δcp0.5h = CP0.5-FCP. Glucagon levels (GLA0, GLA0.5 h, GLA1h, GLA2h, GLA3h) on fasting basis, 0.5 h, 1 h, 2 h and 3 h after the bread meal, and AUC for glucagon in 3 h (AUC_gla3h_) were used to evaluate the pancreatic alpha cell function.

### Statistical analyses

Statistical analysis was performed by SPSS22.0. Data were expressed as median and range for skewed distribution and mean ± standard deviation for normal distribution. One-way ANOVA or Kruskal–Wallis test was applied to compare differences among groups. Spearman’s coefficient determined the relationships between variables. After adjustments for confounding factors, partial correlation coefficient was done to further clarify the relationship between OCN and islet function. Multiple regression analysis showed the independent factors to islets function. *P* < 0.05 was identified as statistical significance.

## Results

### General clinical characteristics of all the included cases

204 patients with type 2 diabetes were included in the study. There were 140 men and 64 women, 31% of women and 69% of men. The average age and diabetes duration of all the participants were 55.06 ± 14.0 years, and 6.0 (2.0,12.75) years. The BMI of all patients was 25.28 (23.20,27.16).

### Differences in serum OCN stratified by sex, BMI, age, diabetes duration, HbA1C

**Fig. 1:**
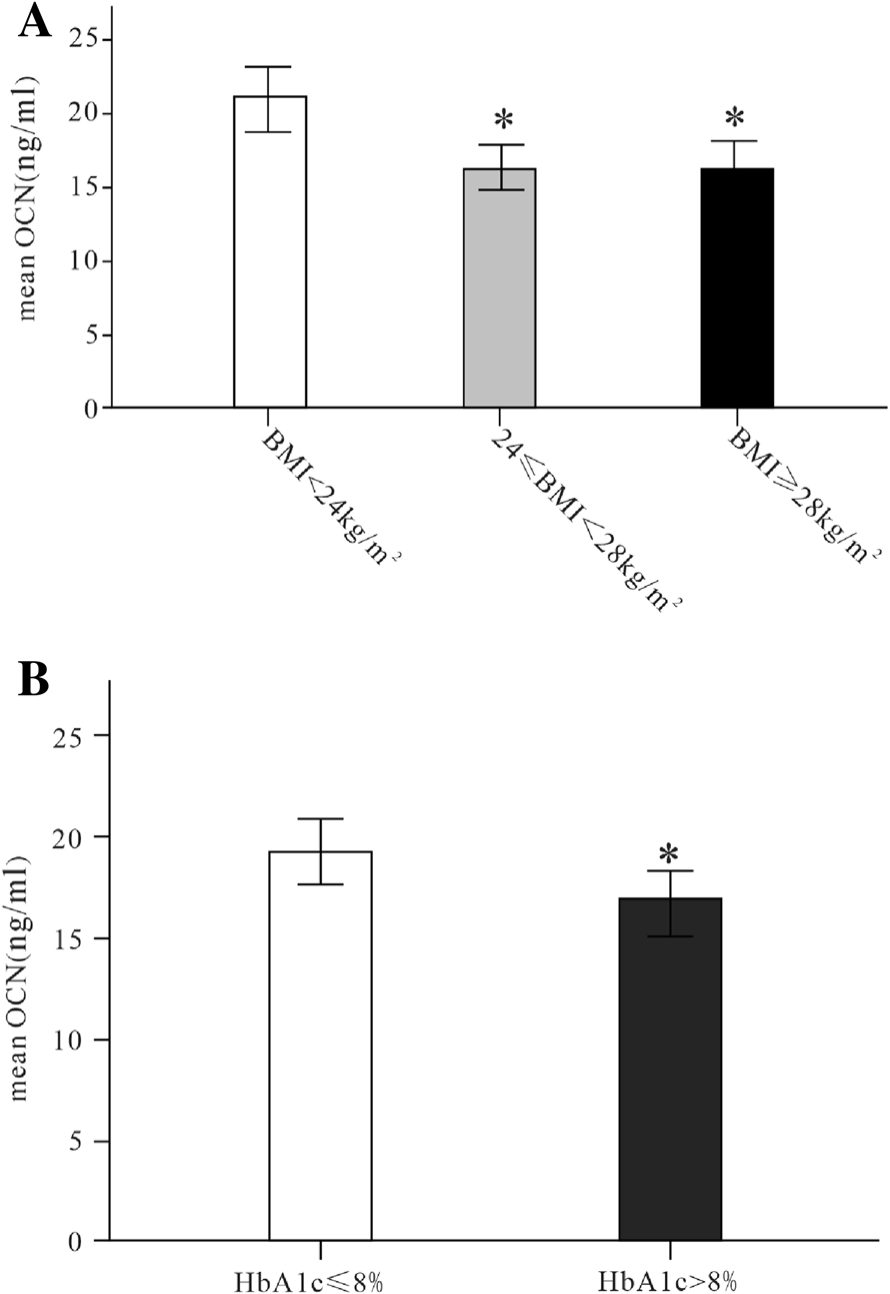
The level of serum osteocalcin was the highest in normal weight BMI < 24 kg/m^2^ group, and the levels of serum osteocalcin in BMI ≥ 28 kg/m^2^ group and 24 kg/m^2^ ≤ BMI < 28 kg/m^2^ group were significantly lower than that in BMI < 24 kg/m^2^ group (respectively, P < 0.05) (**A**). The serum osteocalcin level in patients with HbA1C > 8% was lower than that in patients with HbA1C ≤ 8% (P < 0.05) (**B**). *OCN* Osteocalcin, *BMI* body mass index, *HbA1c* hemoglobin A1c

Divided by sex (Male group and female group), age (youth group (18–44 years old), middle-aged group (45–59 years old), and elderly group (≥ 60 years old)) and diabetes duration (diabetes duration ≤ 5 years and diabetes duration > 5 years), there was no difference in osteocalcin levels between the groups (respectively, *p* > 0.05). Grouped by BMI, serum OCN in BMI < 24 kg/m^2^ Group was significant higher than 24 kg/m^2^ ≤ BMI < 28 kg/m^2^ group and BMI ≥ 28 kg/m^2^ group (respectively, *p* < 0.05) (Fig. 1A). A significantly higher level of serum OCN was observed in the HbA1c ≤ 8 group than it in HbA1c > 8 group (p < 0.05) (Fig. 1B).

### Comparison of baseline clinical parameters of patients with T2DM among tertiles of OCN levels

As Table 1 shows, BMI was lower in T3 group (OCN ≥ 19.78 ng/mL) than group T1 (OCN < 14.02 ng/mL) (25.97 (22.66, 26.69) vs. 26.03 (24.65, 28.08) *p* < 0.05). Serum uric acid (SUA) is lower in T2 group (14.02 ≤ OCN < 19.78 ng/mL) than group T1 (299.08 ± 76.35 vs 340.91 ± 104.92 *p* < 0.05). There were no significant differences in gender composition, age, diabetes duration, systolic blood pressure (SBP), diastolic blood pressure (DBP), alanine aminotransferase (ALT), aspartate aminotransferase AST, urea nitrogen (UN), creatinine (Cr), total cholesterol (TC), triglycerides (TG), high-density lipoprotein cholesterol (HDL), low-density lipoprotein cholesterol (LDL), HbA1c among three groups (respectively, *p* > 0.05) (Table 1).

**Table 1:** Data were stratified according to the tertiles of serum total osteocalcin and presented as means ± SD or median (interquartile range) as appropriate

	T1	T2	T3	p
	< 14.02 ng/mL	14.02–19.78 ng/mL	≥ 19.78 ng/mL	
n	67	68	69	
Male/Female	47/20	51/17	42/27	0.194
Age (years)	55.84 ± 13.00	52.25 ± 14.57	56.61 ± 14.05	0.151
BMI (kg/m^2^)	26.03 (24.65,28.08)	25.97 (23.16,27.04)	25.97 (22.66,26.69)^a^	0.030^*^
Duration (years)	8.0 (2.00,13.00)	4.5 (1.16,12.00)	6.0 (2.00,12.00)	0.483
SBP (mmHg)	133.88 ± 12.59	132.10 ± 12.13	135.70 ± 14.58	0.281
DBP (mmHg)	78.97 ± 10.12	78.26 ± 9.67	24.73 ± 26.88	0.822
ALT (U/L)	28.85 ± 18.33	20.63 ± 11.12	24.73 ± 26.88	0.059
AST (U/L)	24.31 ± 18.64	18.91 ± 9.00	20.33 ± 11.45	0.060
UN (mmol/L)	5.88 ± 1.67	5.57 ± 1.11	5.90 ± 2.51	0.530
Cr (umol/L)	63.56 ± 19.73	58.45 ± 12.72	63.36 ± 23.92	0.222
SUA (umol/L)	340.91 ± 104.92	299.08 ± 76.35^b^	319.68 ± 106.15	0.045^*^
TC (mmol/L)	4.51 ± 1.44	4.97 ± 1.10	4.73 ± 1.05	0.091
TG (mmol/L)	2.30 ± 2.38	1.97 ± 1.66	2.04 ± 1.70	0.581
HDL (mmol/L)	1.02 ± 0.27	1.07 ± 0.24	1.03 ± 0.24	0.564
LDL (mmol/L)	2.64 ± 0.98	2.67 ± 0.74	2.65 ± 0.84	0.974
HbA1C (%)	8.65 ± 2.07	9.12 ± 2.13	8.59 ± 3.16	0.410
FBG (mmol/L)	8.21 ± 2.79	9.30 ± 5.16	7.57 ± 3.11^c^	0.030^*^
PBG0.5h	11.35 ± 3.23	11.83 ± 3.52	10.66 ± 3.96	0.165
PBG1h	14.79 ± 3.74	15.30 ± 4.93	13.75 ± 3.42	0.083
PBG2h	16.64 ± 4.05	16.71 ± 5.36	15.02 ± 3.82^a, c^	0.047^*^
PBG3h	14.83 ± 4.85	14.67 ± 4.57	13.25 ± 4.47	0.095
FINS (mIU/L)	11.8(5.01,18.0)	7.29(2.58,13.3)	7.75(3.27,12.9)	0.045^*^
INS0.5h	17.60(9.8,23.6)	12.45(5.50,24.65)	12.2(7.42,19.90)	0.174
INS1h	25.90(13.2,37.3)	16.15(9.11,36.30)	17.55(11.0,32.7)	0.120
INS2h	29.8(17.6,51.9)	19.8(10.95,46.97)	19.9(9.50,37.27)	0.077
INS3h	27.0(15.90,48.2)	18.35(8.88,37.65)	18.1(11.7,35.12)^a^	0.048^*^
FCP (ng/mL)	1.85(1.20,2.74)	1.47(0.89,2.31)	1.26(0.76,2.29)^a^	0.020^*^
CP0.5h	2.35(1.71,3.81)	1.89(1.27,3.04)	2.20(1.07,2.89)	0.070
CP1h	3.48(2.28,6.07)	2.65(1.65,4.41)	3.46(1.61,4.60)	0.110
CP2h	4.53(3.10,8.15)	4.17(2.49,6.27)	4.34(2.06,6.16)	0.237
CP3h	4.42(3.12,8.09)	3.73(2.31,5.37)	4.42(2.27,6.35)	0.062
GLA0 (pmol/L)	46.44 ± 19.45	46.91 ± 17.30	45.28 ± 17.76	0.865
GLA0.5h	43.76 ± 19.09	41.51 ± 15.82	35.38 ± 17.90^a,c^	0.018^*^
GLA1h	38.32 ± 17.03	35.80 ± 12.93	32.12 ± 12.80^a^	0.042^*^
GLA2h	35.12 ± 18.14	31.87 ± 13.34	30.41 ± 14.60	0.196
GLA3h	30.08 ± 16.03	28.28 ± 12.97	29.10 ± 13.72	0.766
HOMA-β	96.25 ± 183.46	73.72 ± 121.41	79.19 ± 106.41	0.626
HOMA-IR	4.76 ± 4.15	6.37 ± 13.21	4.00 ± 8.16	0.318
AUC_glu3h_	42.60 ±c10.56	43.66 ± 13.00	38.11 ± 11.22^a,c^	0.014^*^
AUC_ins3h_	78.48 (44.12,123.72)	52.40 (26.21,105.52)	53.45 (29.05,93.65)^a^	0.024^*^
AUC_cp3h_	11.59 (7.76,19.68)	9.23 (6.13,13.46)	10.46 (5.14,15.17)	0.127
AUC_gla3h_	113.52 ± 45.21	105.85 ± 31.16	99.08 ± 36.75	0.090
Δcp0.5h	0.36 (− 0.01,0.80)	0.24 (0.06,0.62)	0.53 (0.14,1.06)^a,c^	0.030^*^

Values with differing superscript letters (a, b, c) denoted statistically significant differences across the tertiles. The a or b represented a significant difference compared with T1 group, and the c represented a significant difference compared with T2 group. The * represented significant difference

*OCN* Osteocalcin, *BMI* body mass index, *SBP* systolic blood pressure, *DBP* diastolic blood pressure, *ALT* alanine aminotransferase, *AST* aspartate aminotransferase, *UN* urea nitrogen, *Cr* creatinine, *SUA* Serum uric acid, *TG* triglycerides, *TC* total cholesterol, *HDL* high-density lipoprotein cholesterol, *LDL* low-density lipoprotein cholesterol, *HbA1c* hemoglobin A1c, *FBG* fasting blood-glucose, *PBG* postprandial blood glucose, *FINS* fasting insulin, *INS* postprandial insulin, *FCP* fasting c-peptide, *CP* postprandial c-peptide, *GLA* glucagon, *HOMA-β* Homeostasis model of β cell function, *HOMA-IR* Homeostatic model of insulin resistance, *AUCglu3h* area under the curve of glucose, *AUCins3h* area under the curve of insulin, *AUCgla3h* area under the curve of glucagon, *AUCcp3h* area under the curve of c-peptide, *Δcp0.5h* early postprandial secretion function of islet β cells

### Comparison of glucose metabolism and islet alpha cell and beta cell secretory function among tertiles of OCN levels

#### Glucose metabolism

**Fig. 2:**
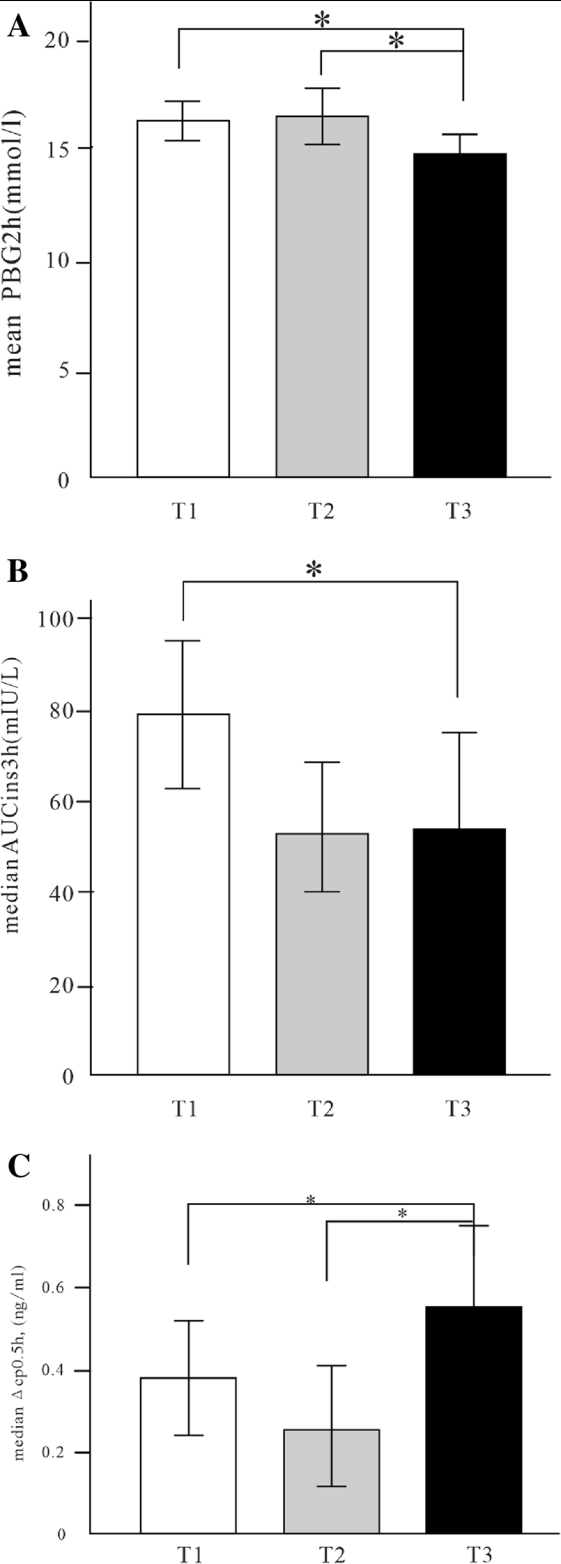

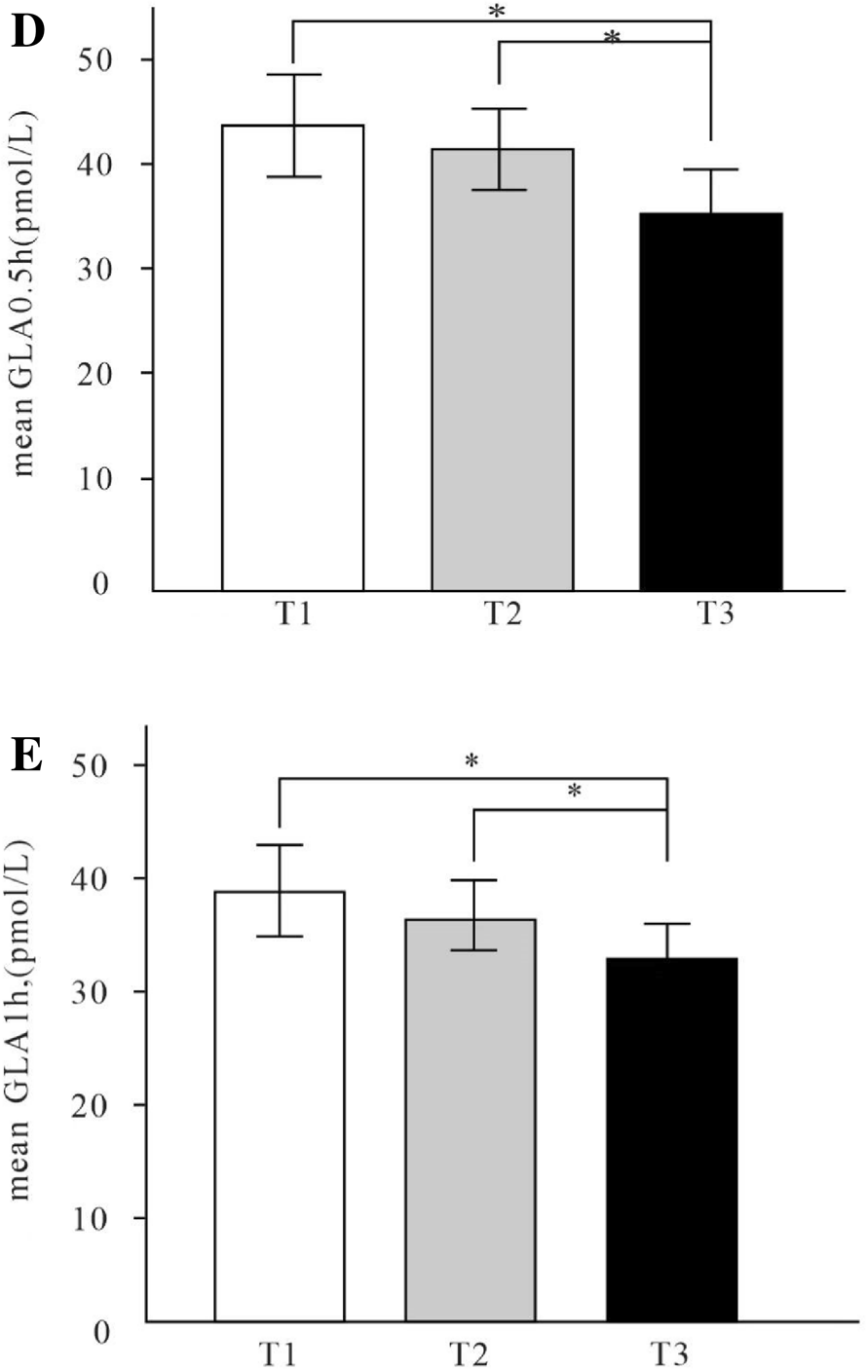
Comparison of glucose metabolism and islet alpha cell and beta cell secretory function among tertiles of OCN levels. PBG2h was lower in T3 group than group T2 and group T1 (15.02 ± 3.82 vs. 16.71 ± 5.36 vs. 16.64 ± 4.05, p < 0.05) (**A**). AUC_ins3h_ in T3 Group was significantly lower than T1 Group (53.45 (29.05, 93.65) vs. 78.48 (44.12, 123.72), p < 0.05) (**B**). Δcp0.5 h in T3 Group was significantly higher than T1Group and T2Group (0.53 (0.14, 1.06) vs. 0.36 (− 0.01, 0.80) vs. 0.24 (0.06, 0.62), p < 0.05) (**C**). GLA0.5 h in T3 group was lower than T2 and T1 groups (35.38 ± 17.90 vs. 41.51 ± 15.82 vs. 43.76 ± 19.09, p < 0.05) (**D**). Compared with the T1 group, GLA1h in the T3 group was lower (32.12 ± 12.80 vs. 38.32 ± 17.03, p < 0.05) (**E**). *OCN* Osteocalcin, *PBG2h* postprandial 2 h blood glucose, *GLA0.5h* postprandial 0.5 h glucagon, *GLA1h* postprandial 1 h glucagon, *AUCins3h* area under the curve of insulin, *Δcp0.5h* early postprandial secretion function of islet β cells

As Table 1 shows, FBG was lower in T3 group than group T2 (7.57 ± 3.11 vs. 9.30 ± 5.16; *p* < 0.05). PBG2h and AUC_glu3h_ was lower in T3 group than group T2 and group T1 (15.02 ± 3.82 vs. 16.71 ± 5.36 vs. 16.64 ± 4.05; 38.11 ± 11.22 vs. 43.66 ± 13.00 vs. 42.60 ± 10.56, *p* < 0.05) (Fig. 2A). There was no significant difference in HOMA-IR among three groups.

#### Beta cell secretory function

There were marked differences in the FINS, INS3h, FCP, AUC_ins3h_, Δcp0.5h among three groups (respectively, *p* < 0.05). Make pairwise comparison of those indexes and discover that the INS3h, FCP, AUC_ins3h_ in T3 Group were significantly lower than T1 Group (18.1 (11.7, 35.12) vs. 27.0 (15.90, 48.2); 1.26 (0.76,2.29) vs. 1.85 (1.20,2.74); 53.45 (29.05,93.65) vs. 78.48 (44.12,123.72), *p* < 0.05), while Δcp0.5h in T3 Group were significantly higher than T1 Group and T2 Group (0.53 (0.14,1.06) vs. 0.36 (− 0.01,0.80) vs. 0.24 (0.06,0.62), p < 0.05). There were no significant differences in HOMA-β, INS0.5h, INS1h, INS2h, CP0.5h, CP1h, CP2h, CP3h, AUC_cp3h_ among three groups. (Table 1 and Fig. 2B, C).

#### Alpha cell secretory function

There were significant differences in GLA0.5h and GLA1h in different tertiles of OCN after a 100 g bread meal load (respectively, *p* < 0.05). Compared with the T1 group, GLA1h in the T3 group was lower (32.12 ± 12.80 vs. 38.32 ± 17.03, *p* < 0.05). GLA0.5h in T3 group was lower than T2 and T1 groups (35.38 ± 17.90 vs. 41.51 ± 15.82 vs. 43.76 ± 19.09, p < 0.05). There were no significant differences in GLA0, GLA2h, GLA3h, AUC_gla3h_ among three groups. (Table 1and Fig. 2D, E).

### Correlation between serum OCN and metrics of glucose metabolism

Spearman’s correlation test (Table 2) revealed that serum OCN was inversely correlated with BMI, ALT, PBG2h (*r*_*s*_ = − 0.190, − 0.212, − 0.141, respectively, *p* < 0.05). Serum OCN was also inversely correlated with FINS, FCP, AUC_ins3h_ (*r*_*s*_ = − 0.138, − 0.198, − 0.146, respectively, *p* < 0.05). Meanwhile, Serum OCN was inversely correlated with alpha cell secretory function indexes such as GLA0.5h, GLA1h, AUC_gla3h_ (*r*_*s*_ = − 0.217, − 0.151, − 0.150, respectively, *p* < 0.05). The test also explored that serum OCN was inversely correlated with HOMA-IR (*r* = − 0.153, *p* < 0.05) (Table 2). Relationship between serum OCN and FCP, GLA0.5h, GLA1h, AUC_gla3h_ (*r* = − 0.160, − 0.211, − 0.153, − 0.151, respectively, *p* < 0.05) remained significant after adjustment for age, sex, the disease durations, BMI and serum UN, serum Cr, SUA, ALT, AST by partial correlation analysis (Table 3).

**Table 2:** Spearman’s correlation test, Correlation between serum OCN and metrics of glucose metabolism

		BMI	ALT	PBG2h	FINS	FCP
OCN	r	− 0.190	− 0.212	− 0.141	− 0.138	− 0.198
	p	0.006	0.002	0.046	0.049	0.004

		GLA0.5 h	GLA1h	HOMA-IR	AUC_gla3h_	AUC_ins3h_
OCN	r	− 0.217	− 0.151	− 0.153	− 0.150	− 0.146
	p	0.002	0.032	0.029	0.032	0.037

*OCN* Osteocalcin, *BMI* body mass index, *ALT* alanine aminotransferase, *PBG2h* postprandial 2 h blood glucose, *FINS* fasting insulin, *FCP* fasting c-peptide, *GLA0.5h* postprandial 0.5 h glucagon, *GLA1h* postprandial 1 h glucagon, *HOMA-IR* Homeostatic model of insulin resistance, *AUCins3h* area under the curve of insulin, *AUCgla3h* area under the curve of glucagon

**Table 3:** partial correlation analysis after adjustment for BMI and ALT.

Control variables			FCP	GLA0.5 h	GLA1h	AUC_gla3h_
BMI&ALT	OCN	r	− 0.160	− 0.211	− 0.153	− 0.151
		p	0.029	0.004	0.037	0.034

*OCN* Osteocalcin, *BMI* body mass index, *ALT* alanine aminotransferase, FCP fasting c-peptide, GLA0.5h postprandial 0.5 h glucagon, GLA1h postprandial 1 h glucagon, *AUCgla3h* area under the curve of glucagon

### Multiple regression analysis showing relationship between the serum OCN and pancreatic secretion

To further determine which variables were independently associated with pancreatic secretion, multiple regression analysis was performed in all patients with type 2 diabetes. The results showed that: (1) OCN and UN were independent contributors to Δcp0.5h (β = 0.035, − 0.203, respectively, p < 0.05), and the other variables such as sex, age, BMI, duration of diabetes, SBP, DBP, ALT, AST, Cr, SUA, TG, TC, HDL, LDL, HbA1c, FBG and PBG0.5h failed to enter the final model; (2) OCN, age and ALT were independent contributors to GLA0.5h (β = − 0.430, 0.239, 0.274, respectively, p < 0.05),and the other variables such as sex, BMI, duration of diabetes, SBP, DBP, AST, UN, Cr, SUA, TG, TC, HDL, LDL, HbA1c, FBG and PBG0.5h failed to enter the final model; (3) OCN was independent contributor to GLA1h (β = − 0.305, p < 0.05), and the other variables such as sex, age, BMI, duration of diabetes, SBP, DBP, ALT, AST, UN, Cr, SUA, TG, TC, HDL, LDL, HbA1c, FBG,PBG0.5h and PBG1h failed to enter the final model (Table 4).

**Table 4:** Multiple regression analysis showing relationships between the serum OCN and pancreatic secretion

Independent variables	β	Sd.E	Standardizedβ	t	p
Constant	61.145	21.839		2.800	0.006
age	0.239	0.118	0.186	2.030	0.044
ALT	0.274	0.114	0.311	2.401	0.017
OCN	− 0.430	0.175	− 0.195	− 2.463	0.015
Dependent variable: GLA0.5h					

Independent variables	β	Sd.E	Standardizedβ	t	p
Constant	34.525	15.596		2.214	0.028
OCN	− 0.305	0.135	− 0.171	− 2.260	0.025
Dependent variable: GLA1h					

Independent variables	β	Sd.E	Standardizedβ	t	p
UN	− 0.203	0.100	− 0.202	− 2.037	0.043
OCN	0.035	0.017	0.151	1.998	0.047
Dependent variable: Δcp0.5h					

*OCN* Osteocalcin, *ALT* alanine aminotransferase, *GLA0.5h* postprandial 0.5 h glucagon, *GLA1h* postprandial 1 h glucagon, *UN* urea nitrogen, *Δcp0.5h* early postprandial secretion function of islet β cells

## Discussion

A growing number of clinical studies have observed that osteocalcin is associated with blood glucose control and metabolic syndrome [5, 7]. Our study firstly explored the relationship between serum osteocalcin and islet function, especially glucagon secretion in islet ɑ cells.

In the present cross-sectional study most of the patients were overweight or obese type 2 diabetes, and insulin resistance is significant in this population. There were no significant differences in serum osteocalcin levels among groups by sex, age and duration of diabetes. This was different from previous studies that serum osteocalcin may be affected by gender and age [9, 36], and may be related to the difference of the selected population.

Serum osteocalcin in normal weight patients with type 2 diabetes was significantly higher than in overweight or obese patients with type 2 diabetes. The serum osteocalcin level was significantly negatively correlated with BMI and HOMA-IR. The relationship between BMI and OCN has been confirmed by multiple studies in different populations. A meta-analysis of 28 cross-sectional studies found an overall significant inverse association between serum osteocalcin and BMI in healthy adults, especially in metabolic syndrome patients [6]. Moreover, osteocalcin concentration in obese and overweight people is lower than normal controls, this is in agreement with other studies on postmenopausal women [5, 22]. A mean of 16.8% weight loss led to increases in OCN levels, and an 8.7% weight loss plus regular exercise led to increased circulating OCN that paralleled reduced fat mass [37].

Similar to previous studies [20, 38, 39], we also found a significant positive association between serum osteocalcin levels and glycemic control. Patients with Type 2 diabetes mellitus with higher serum osteocalcin level had better blood glucose control (HbA1C ≤ 8%), especially lower fasting blood glucose and 2 h postprandial blood glucose. In the highest serum OCN level group the area under the blood glucose curve of standard steamed bread meal experiment in 3 h decreased significantly.

In the highest serum OCN levels group, fasting insulin, postprandial plasma insulin at the third hour and area under 3-h insulin curve of 100 g standard bread experiment were significantly reduced, as well as FCP. Therefore, this reduced fasting, postprandial and total insulin secretion, alleviated hyperinsulinemia in overweight or obese type 2 diabetes. Conversely, early pancreatic beta cell secretion, Δcp0.5h, was significantly increased in the highest OCN group, and multiple regression analysis showed that serum OCN was independent contributors to Δcp0.5h. This is consistent with the results of previous studies [4, 25, 26, 28, 40, 41]. Osteocalcin treatment increased insulin release, and OCN level was negatively correlated with fasting plasma glucose, HbA1c, insulin resistance, BMI and body fat. In our study Osteocalcin alleviated insulin resistance, reduced hyperinsulinemia and enhanced insulin action in type 2 diabetes patients. Excitingly, Osteocalcin increases early postprandial insulin secretion, which contributes to the stability of postprandial blood glucose. More importantly, our research found that patients with Type 2 diabetes mellitus with the highest OCN levels had lowest serum glucagon at postprandial 0.5 h and 1 h. Moreover, osteocalcin was significantly negatively correlated with glucagon secretion at postprandial 0.5 h and 1 h, and especially the total glucagon secretion of 3 h in 100 g bread meal experiment. In particular, multiple regression analysis showed the serum OCN was independent contributor to GLA0.5h and GLA1h. Insulin resistance attenuates the inhibitory effect of insulin on islet ɑ cells secretion and cause hyperglucagonemia, while hyperglucagonemia is an important part of the pathogenesis of insulin resistance in type 2 diabetes [42–46]. Failure to adequately suppress glucagon could lead to the impaired glucose regulation in pre-diabetes and type 2 diabetes [46, 47]. Inappropriate glucagon secretion after meal, which causes a large amount of liver glucose output even after meals, is an important cause of hyperglycemia in type 2 diabetes mellitus, and appropriately suppression of postprandial glucagon secretion is beneficial to postprandial blood glucose control [45, 46]. During last few decades, inhibition of glucagon secretion or its action to improve insulin sensitivity and decrease insulin resistance is one of numerous therapeutic approaches to treat type 2 diabetes mellitus [48]. But agents that inhibit postprandial glucagon secretion or antagonize glucagon action or inhibit glucagon receptor GCGRs have obvious side effects, such as hepatic steatosis, hyperlipidemia, and hyperaminoacidemia, could lead to more serious metabolic disorders and even serious cardiovascular diseases [49–51]. Inhibition of early postprandial glucagon secretion is especially important for blood glucose stabilization [46]. Our study found serum osteocalcin levels were significantly associated with early postprandial glucagon inhibition, such as GLA0.5h and GLA1h. Therefore, this may provide a new appropriate direction for the treatment of type 2 diabetes mellitus. To our best knowledge, for the first time this study evaluated the association of bone formation marker Osteocalcin with the islet ɑ cells secreting glucagon in adult patients with type 2 diabetes mellitus. Whether pancreatic islet ɑ cells also have osteocalcin receptors, and whether the mechanism that osteocalcin acts on pancreatic islet ɑ cells is as the same as it acts on pancreatic islet β cells, still need more in-depth researches.

Our study still has certain limitations. This is only a cross-sectional observational study. The number of cases included is not enough, and all participants are inpatients, so there may be deviations in the study results. Prospective studies can be carried out in the later recruiting a wider population to further clarify the effects of serum osteocalcin on islet α cells secretion function and treatment of diabetes, further more cytological experiments will be done to explore the specific action mechanism of osteocalcin on islet α cells.

## Conclusion

In summary, this research has showed higher serum OCN level is closely related to better blood glucose control, higher insulin sensitivity, increased early-phase insulin secretion of islet β cells and appropriate inhibition of postprandial glucagon secretion of islet ɑ cells in adult patients with type 2 diabetes mellitus.

## Data Availability

The datasets used and/or analyzed during the current study are available from the corresponding author on reasonable request.
